# Joint Fault Diagnosis of IGBT and Current Sensor in LLC Resonant Converter Module Based on Reduced Order Interval Sliding Mode Observer

**DOI:** 10.3390/s24248077

**Published:** 2024-12-18

**Authors:** Xi Zha, Wei Feng, Xianfeng Zhang, Zhonghua Cao, Xinyang Chen

**Affiliations:** 1 Chongqing Academy of Agricultural Sciences, Chongqing 401329, China; cassiezha@163.com (X.Z.); cqnky_zxf@163.com (X.Z.); 13996227484@163.com (Z.C.); 2 School of Electrical Engineering and Automation, Hefei University of Technology, Hefei 230002, China; 18624378373@163.com

**Keywords:** LLC resonant converter, current sensor (CS), IGBT open circuit (OC) fault, adaptive sliding mode observer

## Abstract

LLC resonant converters have emerged as essential components in DC charging station modules, thanks to their outstanding performance attributes such as high power density, efficiency, and compact size. The stability of these converters is crucial for vehicle endurance and passenger experience, making reliability a top priority. However, malfunctions in the switching transistor or current sensor can hinder the converter’s ability to maintain a resonant state and stable output voltage, leading to a notable reduction in system efficiency and output capability. This article proposes a fault diagnosis strategy for LLC resonant converters utilizing a reduced-order interval sliding mode observer. Initially, an augmented generalized system for the LLC resonant converter is developed to convert current sensor faults into generalized state vectors. Next, the application of matrix transformations plays a critical role in decoupling open-circuit faults from the inverter system’s state and current sensor faults. To achieve accurate estimation of phase currents and detection of current sensor faults, a reduced-order interval sliding mode observer has been designed. Building upon the estimation results generated by this observer, a diagnostic algorithm featuring adaptive thresholds has been introduced. This innovative algorithm effectively differentiates between current sensor faults and open switch faults, enhancing fault detection accuracy. Furthermore, it is capable of localizing faulty power switches and estimating various types of current sensor faults, thereby providing valuable insights for maintenance and repair. The robustness and effectiveness of the proposed fault diagnosis algorithm have been validated through experimental results and comparisons with existing methods, confirming its practical applicability in real-world inverter systems.

## 1. Introduction

LLC resonant converters have been widely used in auxiliary power systems of urban rail transit systems such as subways, light rails, and maglev trains due to their simple construction, wide input voltage range, high power density, and easy implementation of soft switching characteristics [1,2,3]. However, due to the complex and variable operating environment of LLC resonant converters, their failure rate remains high, which has become a challenge restricting the development of electric vehicles [4]. When the switching tube fails, it is difficult for the resonant converter to operate near the resonance point while maintaining the output voltage constant using pulse frequency modulation. In addition, CS, as an indispensable feedback component in LLC resonant converter systems, once a fault occurs, it will directly threaten the stability of the entire charging system [5]. Therefore, in-depth exploration and research on fault diagnosis methods for power switch tubes and CS in LLC resonant converter systems have profound significance for ensuring the safe operation of DC charging piles and promoting the healthy development of the electric vehicle industry.

Power switching transistors frequently experience open and short circuit faults. While serious short-circuit faults can typically be isolated using standard protective devices, open circuit faults usually do not cause immediate severe damage [6,7]. However, open-circuit faults can have significant consequences, as prolonged exposure to such conditions may lead to secondary failures in other components, potentially triggering emergency shutdowns in systems like the LLC resonant converter. This critical issue has garnered increasing interest in the research community, particularly regarding the diagnosis of open-circuit faults within these converters. In the realm of fault diagnosis techniques, methods can be broadly classified into two categories [8,9,10,11]. Data-driven approaches leverage advanced signal processing and artificial intelligence algorithms for fault identification; however, these methods typically require substantial amounts of data and high computational power, making them less suitable for real-time online diagnosis. On the other hand, circuit-driven methods focus on techniques based on voltage, current, or model-based analysis. While there has been research aimed at the rapid detection of voltage characteristics, these methods often necessitate additional sampling and diagnostic circuitry. Together, these insights highlight the importance of developing effective fault diagnosis strategies that can operate reliably under real-time constraints while addressing the unique challenges presented by open-circuit faults [12,13,14,15,16]. In contrast, current-based techniques utilize existing signals for diagnosing open circuit faults. Notable methods include the Concordia current mode analysis and average current methods, which have been extensively studied [17]. However, these approaches generally require longer detection times to ensure accuracy.

In LLC resonant converters, control system faults can arise not only from power switch failures but also from the vulnerability of current sensors (CS) to faults. The closed-loop control inherent in high-performance control systems means that any malfunction in the current sensor can lead to erroneous feedback, which may ultimately trigger system shutdowns. As a result, there is a pressing need for rapid diagnosis of current sensor faults to ensure system reliability. Currently, the diagnostic methods for current sensor faults are mainly categorized into model-driven approaches and data-driven approaches, each offering different advantages and challenges in the fault detection process [18,19]. Model-based fault diagnosis methods focus on detecting and analyzing sensor faults by establishing mathematical models and observers of the system. These methods typically have faster diagnostic speeds and lower data requirements. Data-driven fault diagnosis mainly relies on signal processing, artificial intelligence, and other technologies to deeply mine and analyze sensor data, in order to achieve fault diagnosis. As mentioned in reference [18], an LLC converter open circuit fault diagnosis method based on resonant capacitor voltage observation is proposed. This method provides a foundation for fault tolerance by monitoring the voltage changes of resonant capacitors and quickly locating faulty switches. The algorithm introduced in reference [19] is a fault diagnosis method based on the short-time Fourier transform (STFT). This algorithm utilizes the main and auxiliary side currents as feature quantities to perform its analysis. By applying the STFT, it analyzes the frequency components of the current to identify faults effectively. For fault localization, the method employs envelope detection to determine the specific arm of the fault. A significant advantage of this approach is that it does not require additional sensors, thereby reducing overall hardware costs.

The literature review in references [20,21,22] discusses various fault diagnosis methods for current sensors (CS) used in converter control systems. These methods share similarities with open-circuit fault diagnosis techniques for power switches, primarily relying on current analysis or model-driven strategies. Among these approaches, model-driven methods are preferred due to their rapid diagnostic capabilities and lower data requirements. Specific techniques include the proportional full-state observer, as mentioned in reference [23], which is effective for diagnosing current sensor faults and offers quick response times. Additionally, the sliding mode observer, discussed in reference [24], has also been developed for diagnosing current sensor faults across different types of systems.

The above fault diagnosis methods are all for diagnosing faults in a single power electronic device. Due to the fact that the charging module of the DC charging pile also needs to consider the impact of high-frequency transformers on the inverter circuit and rectifier circuit, which is significantly different from the diagnostic objects of the above diagnostic methods, the above-mentioned methods are difficult to directly apply.

Research has shown that the diagnosis of open circuit faults in power switch tubes and current sensor faults has attracted widespread attention. However, existing research often diagnoses the two types of faults separately, ignoring their mutual influence. Two types of faults, open circuit faults and current sensor faults, can both lead to distortion in phase currents and may exhibit similar characteristics in certain situations. This similarity poses a challenge, as open circuit faults can interfere with the diagnosis of current sensor faults, ultimately affecting the accuracy of diagnosing the open circuit faults themselves. To mitigate the risk of misdiagnosis, it is essential to develop a method capable of simultaneously diagnosing both types of faults effectively. In addition, using a single algorithm to handle two types of faults is simpler than using two algorithms, as analysis, calculations, and code can be partially shared. Meanwhile, this method helps to quickly locate faulty equipment and improve maintenance efficiency. Therefore, it is of great significance to develop a method that can quickly and accurately diagnose both power switch open circuit faults and current sensor faults simultaneously.

Current research on the synchronous diagnosis of open-circuit (OC) faults and current sensor (CS) faults in LLC resonant converters is relatively limited. This paper introduces a diagnosis method that utilizes a reduced-order interval sliding mode observer (SMO) to address both types of faults simultaneously. The article also outlines the primary contributions of this proposed method, emphasizing its potential to enhance fault detection in these systems.

(1) We conducted an innovative study on the faults of power switching tubes, specifically open circuit (OC) and current sensor (CS) faults, in LLC resonant converters using a unified approach. The proposed method simplifies fault detection significantly due to the shared fault phase detection algorithm and common fault identification variables compared to employing two separate methods for each fault type. More importantly, this single-method approach reduces the likelihood of false alarms arising from one fault interfering with the other, thereby facilitating easier subsequent maintenance.

(2) A new reduced-order interval sliding mode observer (SMO) has been developed, incorporating an innovative adaptive sliding mode observer approach and reduced steady-state resonance. This estimation method utilizes a convex weighted sum of upper and lower estimates, effectively accounting for uncertain parameters and unknown disturbances affecting the inverter system, leading to accurate and rapid estimation of phase currents. The advantages of this method include enhanced diagnostic speed and robustness, making it a valuable contribution to fault diagnosis in LLC resonant converters.

(3) The proposed fault detection and identification method relies solely on the direct current (DC) side output current. It features a design for detection variables and their corresponding fault diagnosis thresholds, achieving actual fault detection times of less than 1 ms, which ensures both rapid and robust detection results. The robustness and feasibility of this method have been validated by considering factors such as fluctuations in DC side voltage and imbalances in system parameters.

## 2. Model Description

### 2.1. Circuit Model of LLC Resonant Converter for Charging Module

**Figure 1:**
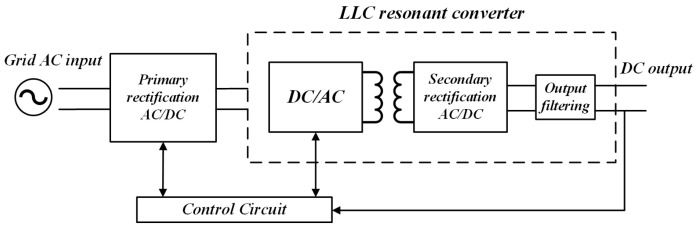
The topology of Charging Module.

Figure 1 shows the two-stage transformation electrical structure of the DC charging station charging module, with the rectifier circuit as its front-end structure, responsible for implementing rectification and active power factor correction. The LLC resonant conversion circuit is the secondary structure responsible for voltage conversion and electrical isolation. Due to the high efficiency of energy conversion achieved by the LLC resonant conversion circuit in the charging module, which directly provides DC input to the load, it is an important component of the charging module. Therefore, this paper mainly studies the CS fault diagnosis method of the LLC resonant conversion circuit.

**Figure 2:**
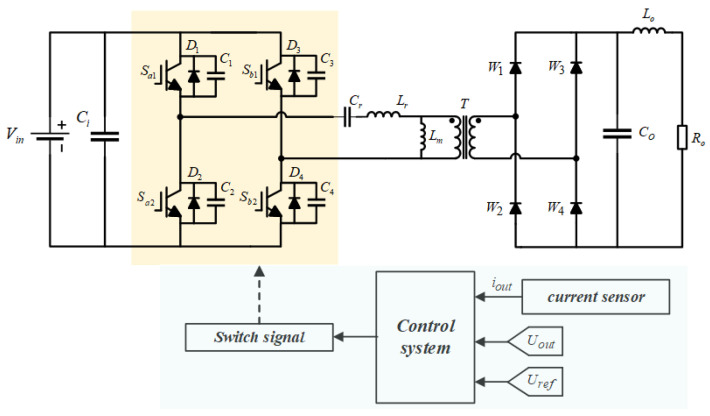
LLC resonant converter circuit topology diagram.

The LLC resonant converter circuit consists of an inverter bridge, a high-frequency transformer *T*, a rectifier bridge, and a filter. A schematic representation of this circuit structure is provided in Figure 2, illustrating the interconnections and layout of its components [25]. The inverter side of the LLC resonant converter circuit is a full bridge, fully controlled inverter circuit. The power modules in the inverter bridge include four switching tubes $S_{a1},S_{a2},S_{b1},S_{b2}$, parasitic capacitors $C_{1},C_{2},C_{3},C_{4}$, and anti-parallel diodes $D_{1},D_{2},D_{3},D_{4}$. Each switching tube is connected in parallel with one parasitic capacitor and one anti-parallel diode. A series resonant inductor and the primary winding of the high-frequency transformer are connected between the midpoint of the bridge arm. The rectifier side is a full bridge uncontrolled rectifier circuit, which consists of four diodes $W_{1},W_{2},W_{3},W_{4}$. The input terminals of the rectifier bridge are connected to the secondary winding of a high-frequency transformer. This transformer has a primary-to-secondary voltage ratio of $v_{s}\left(t\right)$:$u_{s}\left(t\right)$ = 2.5:1. The filtering module comprises a filtering inductor Lf and a filtering capacitor Cf. Additionally, Ro serves as the load for the phase-shifted full-bridge conversion circuit.

**Figure 3:**
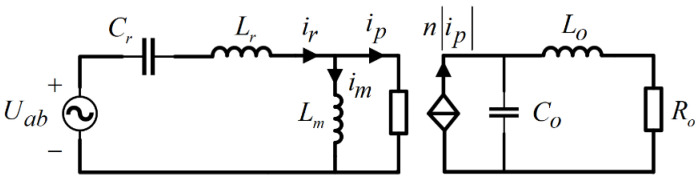
Equivalent diagram of LLC resonant converter circuit.

Figure 3 presents the equivalent circuit diagram for the LLC resonant converter. In this representation, $U_{ab}$ is depicted as a square wave with an amplitude of $\pm V_{in}$, which signifies the equivalent input voltage for the resonant cavity. The term $i_{p}$ denotes the current that traverses the transformer’s primary side. The switching function $S_{AB}={\bar{\mu }}_{a2}\left(\mu_{a1}+{\bar{\mu }}_{a1}{\bar{\delta }}_{a}\right)-{\bar{\mu }}_{b2}\left(\mu_{b1}+{\bar{\mu }}_{b1}{\bar{\delta }}_{b}\right)$ can be formulated as the state equation of the LLC resonant converter, derived from the equivalent circuit and following Kirchhoff’s law:

(1) $$\left\{\begin{array}{c} L_{r}\frac{di_{r}}{dt}+u_{r}+L_{m}\frac{di_{m}}{dt}=u_{AB}\\ L_{m}\frac{di_{m}}{dt}=\mathrm{sgn}\left(i_{p}\right)n\left(L_{0}\frac{di_{o}}{dt}+V_{0}\right)\\ C_{0}\frac{du_{C_{0}}}{dt}=n\left|i_{p}\right|-\frac{L_{0}}{C_{0}R_{0}}\frac{di_{o}}{dt}-\frac{1}{C_{0}R_{0}}V_{0} \end{array}\right.$$

Sort out the LLC resonant converter hybrid logic dynamic model:

(2) $$\begin{array}{c} \left[\begin{array}{c} \frac{di_{o}}{dt}\\ \frac{du_{C_{0}}}{dt} \end{array}\right]=\left[\begin{array}{cc} -\frac{R_{L}}{L_{o}} & 0\\ \frac{1}{C_{0}} & 0 \end{array}\right]\left[\begin{array}{c} i_{o}\\ u_{C_{0}} \end{array}\right]+\left[\begin{array}{cc} 0 & 0\\ \frac{n}{C_{0}} & -\frac{1}{C_{0}R_{L}} \end{array}\right]\left[\begin{array}{c} \left|i_{p}\right|\\ V_{o} \end{array}\right]\\ +\frac{1}{n\mathrm{sgn}\left(i_{p}\right)}\left[\begin{array}{ccc} \frac{S_{AB}}{L_{o}} & -\frac{L_{r}}{L_{o}} & -\frac{1}{L_{o}}\\ -\frac{S_{AB}}{C_{0}R_{L}} & \frac{L_{r}}{C_{0}R_{L}} & \frac{1}{C_{0}R_{L}} \end{array}\right]\left[\begin{array}{c} V_{in}\\ \frac{di_{r}}{dt}\\ u_{r} \end{array}\right] \end{array}$$

where $x=\left[\begin{array}{c} i_{o}\\ u_{C_{0}} \end{array}\right]$, $\varphi =\left[\begin{array}{c} V_{in}\\ \frac{di_{r}}{dt}\\ u_{r} \end{array}\right]$, $\gamma =\left[\begin{array}{c} \left|i_{p}\right|\\ V_{o} \end{array}\right]$, $A=\left[\begin{array}{cc} -\frac{R_{L}}{L_{o}} & 0\\ \frac{1}{C_{0}} & 0 \end{array}\right]$, $\hbar =\frac{1}{n\mathrm{sgn}\left(i_{p}\right)}$, $G=\left[\begin{array}{cc} 0 & 0\\ \frac{n}{C_{0}} & -\frac{1}{C_{0}R_{L}} \end{array}\right]$, $B=\left[\begin{array}{ccc} \frac{S_{AB}}{L_{o}} & -\frac{L_{r}}{L_{o}} & -\frac{1}{L_{o}}\\ -\frac{S_{AB}}{C_{0}R_{L}} & \frac{L_{r}}{C_{0}R_{L}} & \frac{1}{C_{0}R_{L}} \end{array}\right]$. According to the working principle of the LLC resonant converter circuit, the mixed logic dynamic model of the LLC resonant converter circuit can be obtained as follows:

(3) $$\left\{\begin{array}{c} \dot{x}=Ax+\hbar B\varphi +G\gamma \\ y=Cx \end{array}\right.$$

In the formula, $C=diag\left[\begin{array}{cc} 1 & 1 \end{array}\right]$ is the output matrix of the system, and *y* is the output of the system.

#### 2.1.1. Cs Fault Description

In the LLC resonant converter system, the single-phase current measured by CS is used as feedback information to the control system to control the on/off of the power switch tubes in the inverter, converting DC power into AC power and forming a closed-loop control system. Once CS malfunctions, it will have a serious impact on the entire LLC resonant converter system and even lead to system paralysis. Therefore, in order to illustrate the impact of different CS faults on the LLC resonant converter systems, this section will analyze and diagnose typical faults such as CS open circuit, drift, gain faults, and minor faults in current sensors.

The measurement value $i^{\prime }$ represents the readings from the current sensor, while *i* denotes the normal current value. Typical faults associated with the current sensor include open circuit faults, drift faults, and gain faults. Expressions for these faults can be found in reference [26].

The expression for an open circuit fault is:

(4) $$i_{f}\left(t\right)=\left\{\begin{array}{c} i\left(t\right),0<t<t_{0}\\ 0,t\geq t_{0} \end{array}\right.$$

In the interval $0<t<t_{0}$, the function $i_{f}$ is equal to $i\left(t\right)$, indicating that the current sensor (CS) is operating normally, as its measured value corresponds to the expected current. However, at $t\geq t_{0}$, when the CS measurement reads zero, it signifies an open circuit fault in the sensor. This analysis is derived from the previously discussed model; it can be seen that the characteristic of CS open circuit fault is that after CS occurs an open circuit fault, all current information is lost, and the measured value fed back to the control system by CS quickly becomes 0.

The expression for drift fault is:

(5) $$i_{f}\left(t\right)=\left\{\begin{array}{c} i\left(t\right),0<t<t_{0}\\ i\left(t\right)+\varepsilon ,t\geq t_{0} \end{array}\right.$$

In the formula, ε is the drift amount of the drift fault. When ε=0, it indicates that the sensor has not malfunctioned and is in normal working condition. The larger the drift amount ε, the greater the drift amplitude and the greater the degree of fault. $t_{0}$ is the initial moment when the fault occurs, indicating that the sensor has experienced drift faults since then. From the above drift fault model, it can be seen that the characteristic of CS drift fault is the existence of fixed numerical differences based on the normal current signal, that is, the overall increase or decrease of the normal current signal by a certain amount of drift, and the drift amount is constant.

The expression for gain fault is:

(6) $$i_{f}\left(t\right)=\left\{\begin{array}{c} i\left(t\right),0<t<t_{0}\\ k_{i}i\left(t\right),t\geq t_{0} \end{array}\right.$$

In the formula, $k_{i}$ is the gain coefficient of the gain fault. When $k_{i}=1$, it indicates that the sensor has not malfunctioned and is in normal working condition. As the value of $k_{i}$ increases, both the change in gain and the severity of the fault also rise. The point $t_{0}$ marks the onset of the fault, indicating that the sensor began to experience a gain-related issue at that time. According to the gain fault model discussed earlier, a key feature of the CS gain fault is that the phase frequency of the fault current signal matches that of the normal current signal. However, during a gain fault, the amplitude of the fault current varies depending on the gain coefficient, either increasing or decreasing accordingly.

#### 2.1.2. Fault Analysis of Power Switch Tube

The gate switch signals of power switch tube $S_{k1},S_{k2}$ are defined as $\mu_{k1},\mu_{k2}$, and the logic variable δ represents the current flow direction; the variable $\mu_{k1}=1$ signifies that the switch $S_{k1}$ is in a conducting state, while $\mu_{k1}=0$ indicates that it is off. Similarly, $\mu_{k2}=1$ denotes that the switch $S_{k2}$ is conducting, and $\mu_{k2}=0$ means it is turned off. Additionally, the parameter δ=1 represents current flowing into the winding, i.e., i>0, whereas δ=−1 indicates that current is exiting the winding, i.e., i<0.

**Figure 4:**
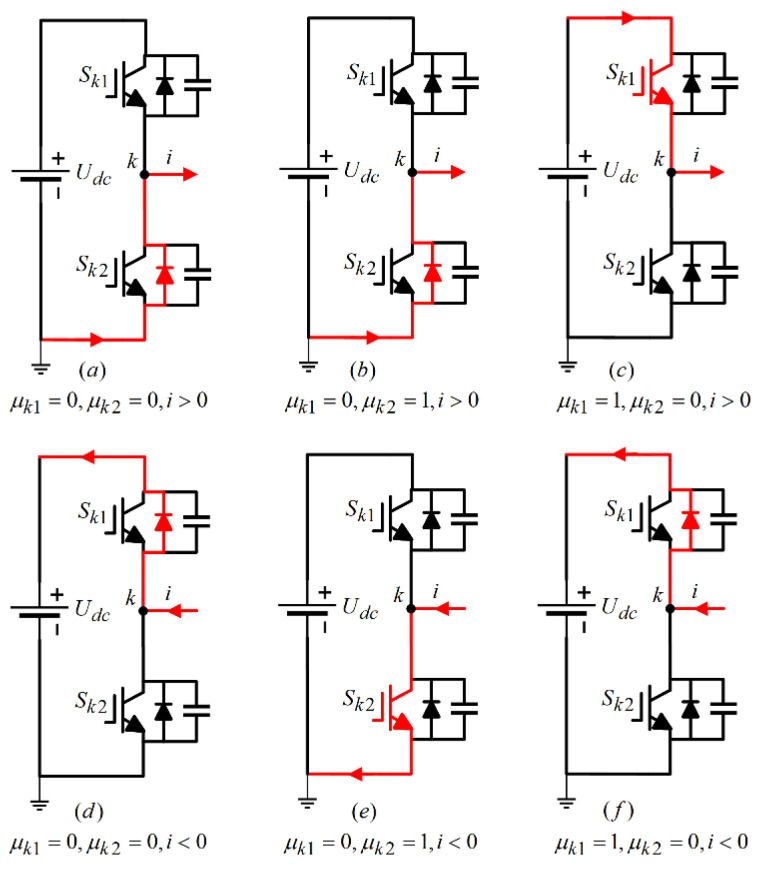
phase-k current flow path under normal working conditions.

The current flow path of the LLC resonant converter system inverter under normal operating conditions is shown in Figure 4. Among them, Figure 4a represents $\mu_{k1}=0,\mu_{k2}=0,i>0$, where all switch tubes $S_{k1},S_{k2}$ are turned off and current flows into the winding. At this time, $u_{ab}=0$; Figure 4b shows $\mu_{k1}=0,\mu_{k2}=1,i>0$, where switch $S_{k1}$ is turned off and switch $S_{k2}$ is turned on, and current flows into the winding. At this time, $u_{ab}=0$; Figure 4c shows $\mu_{k1}=1,\mu_{k2}=0,i>0$, where switch $S_{k1}$ is turned on and switch $S_{k2}$ is turned off, and current flows into the winding. At this point, $u_{ab}=U_{dc}$. Similarly, when i<0, i.e., current flows out of the winding, the analysis of the current flow path is similar.

Through the analysis of the working principle of the switching transistor in the LLC resonant converter system inverter mentioned above, it can be concluded that the switching function of the primary side switching transistor is $\varepsilon_{ab}={\bar{\mu }}_{a2}\left(\mu_{a1}+{\bar{\mu }}_{a1}{\bar{\delta }}_{a}\right)-{\bar{\mu }}_{b2}\left(\mu_{b1}+{\bar{\mu }}_{b1}{\bar{\delta }}_{b}\right)$.

As illustrated in Figure 4, under typical operating conditions, there are three current flow paths regardless of whether the current is entering or exiting the winding. However, when an open circuit fault occurs in the power switch tube, its operational state alters, resulting in a change in the current flow path. Given that both bridge arms share the same structure, this section focuses on analyzing the current flow path and the fault current waveform, specifically when the power switch tube in the first bridge arm encounters an open circuit fault.

**Figure 5:**
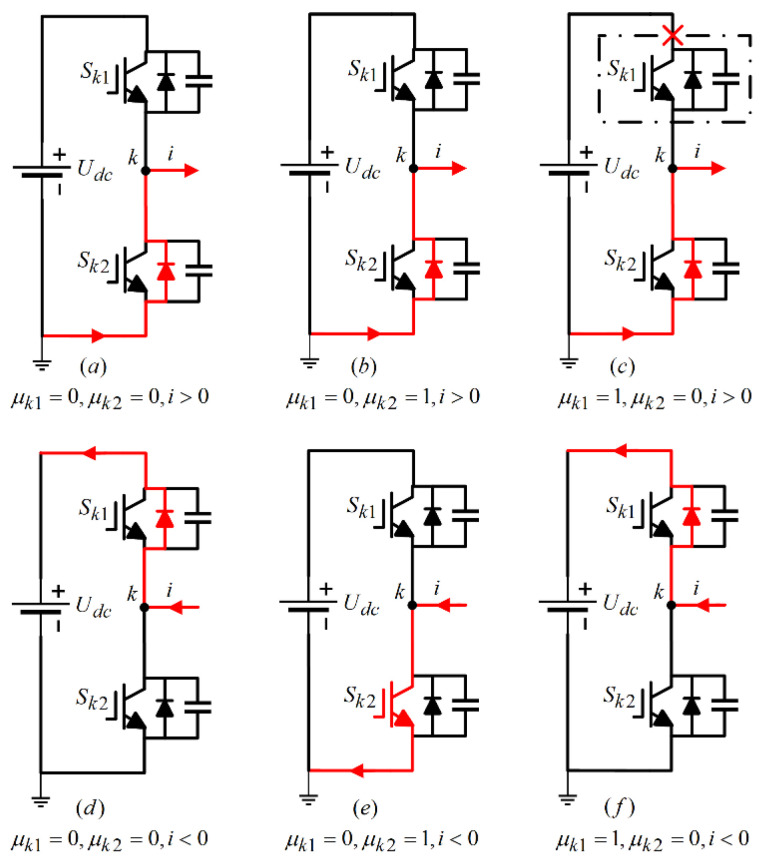
Phase-k current flow path in case of $S_{k1}$ open circuit fault.

Figure 5 illustrates the current flow path for a single-phase circuit when the power switch tube $S_{k1}$ in the first bridge arm is in an open circuit fault state. As observed in Figure 4a,b and Figure 5d,e, following the occurrence of the open circuit fault in $S_{k1}$, the gate signal $\mu_{k1}$ transitions to 0, indicating that switch $S_{k1}$ is off. In this scenario, current does not pass through $S_{k1}$, which means it does not influence the overall current flow path. When the control signal $\mu_{k1}=1$ indicates that switch $S_{k1}$ is conducting, an open circuit fault can lead to two distinct scenarios:

1. If i<0 (current flowing out of the winding), the current will bypass $S_{k1}$ through the freewheeling diode without altering the current flow path, as depicted in Figure 5f.

2. Conversely, if i>0 (current entering the winding), the normal flow would be through $S_{k1}$ in the upper bridge arm. However, because of the open circuit fault in $S_{k1}$, the current flow is obstructed, resulting in a loss of information for the upper half-cycle of the current, effectively leading to an amplitude of zero. This altered current flow path is represented in Figure 5c.

Based on the previous analysis, a model is established by integrating Equation (3). This model takes into account potential unknown disturbances that may arise during operation, such as measurement noise. It is specifically designed to address current sensor faults and open circuit faults in the power switches of the LLC resonant converter system. The detailed expressions of the model will be provided in subsequent equations.

(7) $$\left\{\begin{array}{c} \dot{x}=Ax+\vartheta \hbar B\varphi +G\gamma +Dd+F_{a}f_{a}\\ y=Cx+F_{s}f_{s} \end{array}\right.$$

where $f_{s}$ is the fault of the current sensor, *F* is the coefficient matrix of fault $f_{s}$, *d* is the bounded unknown disturbance, that is, $d^{-}<d<d^{+}$, $F_{a}=I_{2}$, $F_{s}=I_{2}$, *d* are the coefficient matrices of the disturbance. ϑ is an imprecise parameter of the system, representing the inaccuracy of parameter perturbations, internal disturbances, etc. in the system, and its upper and lower bounds are known as $\vartheta \in \left[\vartheta^{-},\vartheta^{+}\right]$, then ϑℏBφ satisfies $\vartheta^{-}\hbar B\varphi <\vartheta \hbar B\varphi <\vartheta^{+}\hbar B\varphi$. From $y=Cx+Ff_{s}+Dd$, it can be seen that the LLC resonant converter system with current sensor faults established by system (7) is directly related to the characteristics of the current sensor.

## 3. Joint Fault Diagnosis Method

**Figure 6:**
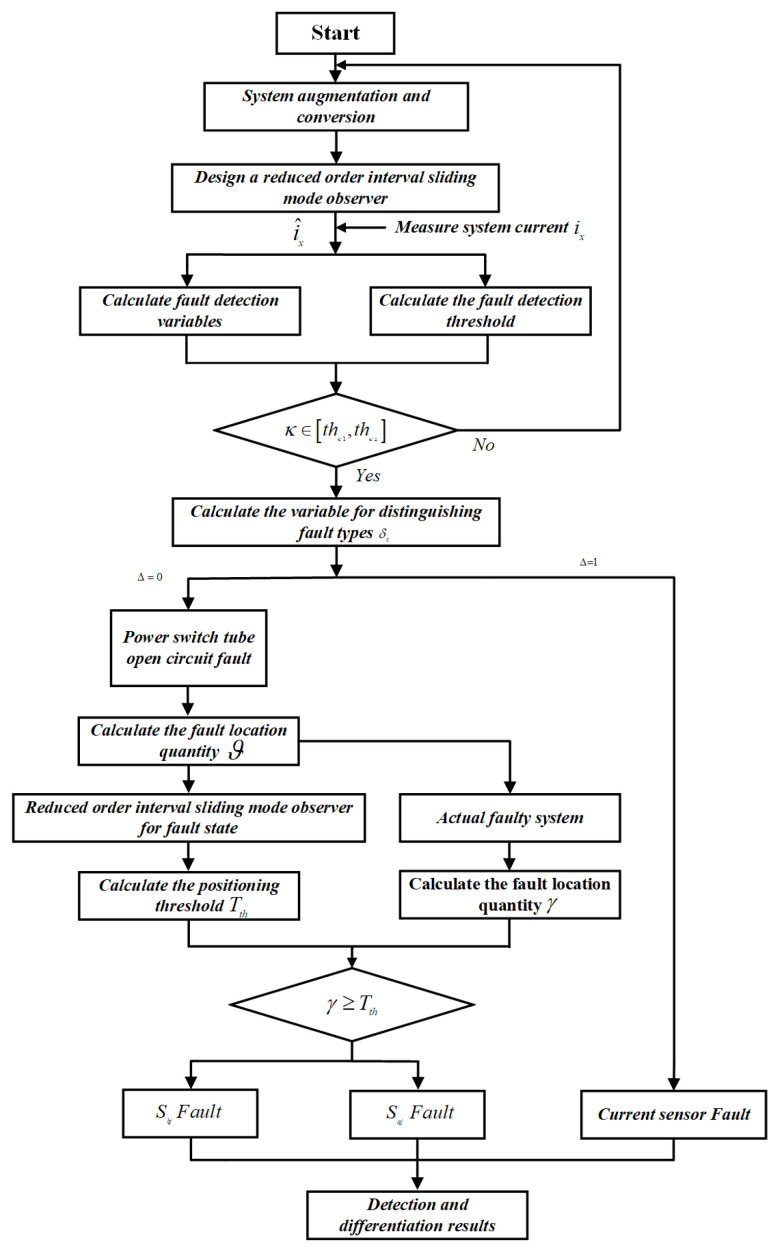
Fault diagnosis process.

An extended system has been established for System (7), which decouples the open circuit faults of the power switches through matrix transformations. Additionally, a reduced-order sliding mode observer has been developed to simultaneously estimate the states of the LLC resonant converter system and the current sensor faults. Based on these estimation results, a fault diagnosis method is proposed as shown in Figure 6.

### 3.1. Coordinate Transformation

To achieve the evolution detection of LLC resonant converter system faults, the state augmentation method is used to augment the mixed logic dynamic model (7) of the system containing small faults in current sensors and power switch tube faults, and the augmented system is obtained as follows:

(8) $$\left\{\begin{array}{c} \bar{H}\dot{\bar{x}}=\bar{A}\bar{x}+\vartheta \hbar \bar{B}\varphi +\bar{G}\gamma +\bar{D}d+\bar{F}f\\ y=\bar{C}\bar{x} \end{array}\right.$$

where $\bar{x}=\left[\begin{array}{c} x\\ ℘ \end{array}\right]$, $\bar{A}=\left[\begin{array}{cc} A & 0\\ 0 & -I_{2} \end{array}\right]$, $\bar{B}=\left[\begin{array}{c} B\\ 0 \end{array}\right]$, $\bar{C}=\left[\begin{array}{cc} C & I_{2} \end{array}\right]$, $\bar{D}=\left[\begin{array}{c} D\\ 0 \end{array}\right]$, $\bar{G}=\left[\begin{array}{c} G\\ 0 \end{array}\right]$, $\bar{H}=\left[\begin{array}{cc} I_{2} & 0\\ 0 & 0 \end{array}\right]$, $f=\left[\begin{array}{c} f_{a}\\ f_{s} \end{array}\right]$, $\bar{F}=\left[\begin{array}{cc} F_{a} & 0\\ 0 & F_{s} \end{array}\right]$, $℘=F_{s}f$.

**Remark 1.**  *The invariant zero point of system $\left(\bar{A},\bar{E},\bar{C}\right)$ is located on the left half plane of plane S.*

To reduce the number of observation variables in System (7), a coordinate transformation matrix has been proposed. The purpose of this transformation is to simplify the observation process of the system.

(9) $${\bar{O}}^{-1}=\left[\begin{array}{cc} O_{1_{2\times 2}} & O_{2_{2\times 2}}\\ O_{3_{2\times 2}} & O_{4_{2\times 2}} \end{array}\right]=\left[\begin{array}{cc} -\frac{1}{2}I_{2} & \frac{1}{2}I_{2}\\ \frac{1}{2}I_{2} & \frac{1}{2}I_{2} \end{array}\right]$$

Afterwards, using matrix transformation ${\bar{x}}^{\left(1\right)}=\bar{O}\bar{x}$, the augmented system (8) is expressed as

(10) H¯1x¯˙1=A¯1x¯1+ϑℏB¯φ+G¯γ+D¯d+F¯fy=C¯1x¯1=x¯1x¯21

where ${\bar{x}}^{\left(1\right)}=\bar{O}\bar{x}=\left[\begin{array}{c} {\left({\bar{x}}_{1}^{\left(1\right)}\right)}^{T}\\ {\left({\bar{x}}_{2}^{\left(1\right)}\right)}^{T} \end{array}\right]$, ${\bar{A}}^{\left(1\right)}=\bar{A}{\bar{O}}^{-1}$, ${\bar{C}}^{\left(1\right)}=\bar{C}{\bar{O}}^{-1}=\left[\begin{array}{cc} 0_{2\times 2} & I_{2} \end{array}\right]$, ${\bar{H}}^{\left(1\right)}=\bar{H}{\bar{O}}^{-1}$.

**Remark 2.**  *State component ${\bar{x}}_{2}^{\left(1\right)}$ can be directly obtained by the observer in system (10). Therefore, only ${\bar{x}}_{1}^{\left(1\right)}$ cannot be directly obtained and needs to be estimated.*

It is important to highlight that ${\bar{H}}^{\left(1\right)}$ possesses a non-standard structure. To facilitate further analysis, we introduce the matrix $\bar{Z}$, which leads to additional modifications of system (10) as outlined below:

(11) $$\bar{Z}=\left[\begin{array}{cc} {\bar{O}}_{1}^{-1} & {\bar{O}}_{2}^{-1}\\ 0_{2\times 2} & I_{2} \end{array}\right]=\left[\begin{array}{cc} -2I_{2} & I_{2}\\ 0 & I_{2} \end{array}\right]$$

Obviously, $\bar{Z}$ is a full rank matrix. Multiply both sides of System (10) by ${\bar{Z}}^{-1}$ to obtain:

(12) H¯2x¯˙1=A¯2x¯1+ϑℏB¯2φ+G¯2γ+D¯2d+F¯2fy=x¯1x¯21

where ${\bar{A}}^{\left(2\right)}={\bar{Z}}^{-1}{\bar{A}}^{\left(1\right)}=\left[\begin{array}{cc} {\bar{A}}_{1}^{\left(2\right)} & {\bar{A}}_{2}^{\left(2\right)}\\ {\bar{A}}_{3}^{\left(2\right)} & {\bar{A}}_{4}^{\left(2\right)} \end{array}\right]$, ${\bar{G}}^{\left(2\right)}={\bar{Z}}^{-1}\bar{G}$, ${\bar{B}}^{\left(2\right)}={\bar{Z}}^{-1}\bar{B}=\left[\begin{array}{c} {\bar{B}}_{1}^{\left(2\right)}\\ {\bar{B}}_{2}^{\left(2\right)} \end{array}\right]$, ${\bar{F}}^{\left(2\right)}={\bar{Z}}^{-1}\bar{F}=\left[\begin{array}{c} {\bar{F}}_{1}^{\left(2\right)}\\ {\bar{F}}_{2}^{\left(2\right)} \end{array}\right]$, ${\bar{H}}^{\left(2\right)}={\bar{Z}}^{-1}{\bar{H}}^{\left(1\right)}$.

Next, we will apply the following decomposition to System (12):

(13) $$\begin{array}{c} \left[\begin{array}{cc} I_{2} & -{\bar{Z}}_{2}^{-1}\\ 0 & I_{2}-{\bar{Z}}_{4}^{-1} \end{array}\right]\left[\begin{array}{c} {\dot{\bar{x}}}_{1}^{\left(1\right)}\\ {\dot{\bar{x}}}_{2}^{\left(1\right)} \end{array}\right]=\left[\begin{array}{cc} {\bar{A}}_{1}^{\left(2\right)} & {\bar{A}}_{2}^{\left(2\right)}\\ {\bar{A}}_{3}^{\left(2\right)} & {\bar{A}}_{4}^{\left(2\right)} \end{array}\right]\left[\begin{array}{c} {\bar{x}}_{1}^{\left(1\right)}\\ {\bar{x}}_{2}^{\left(1\right)} \end{array}\right]+\vartheta \hbar \left[\begin{array}{c} {\bar{B}}_{1}^{\left(2\right)}\\ {\bar{B}}_{2}^{\left(2\right)} \end{array}\right]\varphi +\left[\begin{array}{c} {\bar{G}}_{1}^{\left(2\right)}\\ {\bar{G}}_{2}^{\left(2\right)} \end{array}\right]\gamma \\ +\left[\begin{array}{c} {\bar{D}}_{1}^{\left(2\right)}\\ {\bar{D}}_{2}^{\left(2\right)} \end{array}\right]d+\left[\begin{array}{c} {\bar{F}}_{1}^{\left(2\right)}\\ {\bar{F}}_{2}^{\left(2\right)} \end{array}\right]f \end{array}$$

**Theorem 1.**  *If there exists a positive definite matrix, then the following conditions are satisfied:*

(14) $${\left({\bar{F}}^{\left(2\right)}\right)}^{T}P=J{\bar{C}}^{\left(1\right)}$$

*where $P=\left[\begin{array}{cc} P_{1_{2\times 2}} & P_{2_{2\times 2}}\\ P_{2_{2\times 2}}^{T} & P_{4_{2\times 2}} \end{array}\right]$, then, expand the fault vector f and decouple it from ${\bar{x}}_{1}^{\left(1\right)}$ in system (13).*

*Then, introduce the following matrix M:*

(15) $$M=\left[\begin{array}{cc} I_{2} & P_{1}^{-1}P_{2}\\ 0 & I_{2} \end{array}\right]$$

*Now, satisfying condition (14) and multiplying it by M on both sides of System (13) yields:*

(16) $$\begin{array}{c} \left[\begin{array}{cc} I_{2} & -{\bar{Z}}_{2}^{-1}+P_{1}^{-1}P_{2}\left(I_{2}-{\bar{Z}}_{4}^{-1}\right)\\ 0 & I_{2}-{\bar{Z}}_{4}^{-1} \end{array}\right]\left[\begin{array}{c} {\dot{\bar{x}}}_{1}^{\left(1\right)}\\ {\dot{\bar{x}}}_{2}^{\left(1\right)} \end{array}\right]=\vartheta \hbar \left[\begin{array}{c} {\bar{B}}_{1}^{\left(2\right)}+P_{1}^{-1}P_{2}{\bar{B}}_{2}^{\left(2\right)}\\ {\bar{B}}_{2}^{\left(2\right)} \end{array}\right]\varphi \\ +\left[\begin{array}{cc} {\bar{A}}_{1}^{\left(2\right)}+P_{1}^{-1}P_{2}{\bar{A}}_{3}^{\left(2\right)} & {\bar{A}}_{2}^{\left(2\right)}+P_{1}^{-1}P_{2}{\bar{A}}_{4}^{\left(2\right)}\\ {\bar{A}}_{3}^{\left(2\right)} & {\bar{A}}_{4}^{\left(2\right)} \end{array}\right]\left[\begin{array}{c} {\bar{x}}_{1}^{\left(1\right)}\\ {\bar{x}}_{2}^{\left(1\right)} \end{array}\right]+\left[\begin{array}{c} {\bar{G}}_{1}^{\left(2\right)}+P_{1}^{-1}P_{2}{\bar{G}}_{2}^{\left(2\right)}\\ {\bar{G}}_{2}^{\left(2\right)} \end{array}\right]\gamma \\ +\left[\begin{array}{c} {\bar{D}}_{1}^{\left(2\right)}+P_{1}^{-1}P_{2}{\bar{D}}_{2}^{\left(2\right)}\\ {\bar{D}}_{2}^{\left(2\right)} \end{array}\right]d+\left[\begin{array}{c} {\bar{F}}_{1}^{\left(3\right)}\\ {\bar{F}}_{2}^{\left(2\right)} \end{array}\right]f\\ y={\bar{x}}_{2}^{\left(1\right)} \end{array}$$

**Remark 3.**  *Fault separation has been achieved by effectively enhancing and decoupling the system, completely isolating the switch fault $f_{a}$ from the state vector ${\bar{x}}_{1}^{\left(1\right)}$. This state vector consists solely of the inverter system state x, current sensor fault $f_{s}$, and unknown disturbances d. Notably, open switch faults do not impact the state vector ${\bar{x}}_{1}^{\left(1\right)}$, allowing for accurate estimation of the system state and current sensor faults through the implementation of a system observer, even in the presence of power switch faults.*

### 3.2. Design of Reduced Order Interval Sliding Mode Observer

According to Theorem 1, the following reduced-order interval observer and upper bound observer are constructed for System (16):

(17) $$\left\{\begin{array}{c} {\dot{\ell }}^{+}=\left[{\bar{A}}_{1}^{\left(2\right)}+P_{1}^{-1}P_{2}A_{3}^{\left(2\right)}\right]\ell^{+}+\left[{\bar{A}}_{2}^{\left(2\right)}+P_{1}^{-1}P_{2}A_{4}^{\left(2\right)}\right]y+\left[{\bar{Z}}_{2}^{-1}-P_{1}^{-1}P_{2}\left(I_{2}-{\bar{Z}}_{4}^{-1}\right)\right]\dot{y}\\ +\vartheta^{+}\hbar \left[{\bar{B}}_{1}^{\left(2\right)}+P_{1}^{-1}P_{2}{\bar{B}}_{2}^{\left(2\right)}\right]\varphi +\left[{\bar{G}}_{1}^{\left(2\right)}+P_{1}^{-1}P_{2}{\bar{G}}_{2}^{\left(2\right)}\right]\gamma +\left[{\bar{D}}_{1}^{\left(2\right)}+P_{1}^{-1}P_{2}{\bar{D}}_{2}^{\left(2\right)}\right]d^{+}\\ +K_{1}\left({\bar{x}}^{\left(1\right)}-{\hat{\bar{x}}}^{{\left(1\right)}^{+}}\right)+k_{1}f\left({\bar{x}}^{\left(1\right)}-{\hat{\bar{x}}}^{{\left(1\right)}^{+}}\right)\mathrm{sign}\left({\bar{x}}^{\left(1\right)}-{\hat{\bar{x}}}^{{\left(1\right)}^{+}}\right)\\ {\hat{\bar{x}}}^{{\left(1\right)}^{+}}=\ell^{+}+\left[{\bar{Z}}_{2}^{-1}-P_{1}^{-1}P_{2}\left(I_{2}-{\bar{Z}}_{4}^{-1}\right)\right]y\\ {\hat{\bar{x}}}^{+}={\bar{O}}^{-1}\left[\begin{array}{c} {\hat{\bar{x}}}^{{\left(1\right)}^{+}}\\ y \end{array}\right] \end{array}\right.$$

Lower bound observer:

(18) $$\left\{\begin{array}{c} {\dot{\ell }}^{-}=\left[{\bar{A}}_{1}^{\left(2\right)}+P_{1}^{-1}P_{2}A_{3}^{\left(2\right)}\right]\ell^{-}+\left[{\bar{A}}_{2}^{\left(2\right)}+P_{1}^{-1}P_{2}A_{4}^{\left(2\right)}\right]y+\left[{\bar{Z}}_{2}^{-1}-P_{1}^{-1}P_{2}\left(I_{2}-{\bar{Z}}_{4}^{-1}\right)\right]\dot{y}\\ +\vartheta^{-}\hbar \left[{\bar{B}}_{1}^{\left(2\right)}+P_{1}^{-1}P_{2}{\bar{B}}_{2}^{\left(2\right)}\right]\varphi +\left[{\bar{G}}_{1}^{\left(2\right)}+P_{1}^{-1}P_{2}{\bar{G}}_{2}^{\left(2\right)}\right]\gamma +\left[{\bar{D}}_{1}^{\left(2\right)}+P_{1}^{-1}P_{2}{\bar{D}}_{2}^{\left(2\right)}\right]d^{-}\\ +K_{1}\left({\bar{x}}^{\left(1\right)}-{\hat{\bar{x}}}^{{\left(1\right)}^{-}}\right)+k_{1}f\left({\bar{x}}^{\left(1\right)}-{\hat{\bar{x}}}^{{\left(1\right)}^{-}}\right)\mathrm{sign}\left({\bar{x}}^{\left(1\right)}-{\hat{\bar{x}}}^{{\left(1\right)}^{-}}\right)\\ {\hat{\bar{x}}}^{{\left(1\right)}^{-}}=\ell^{-}+\left[{\bar{Z}}_{2}^{-1}-P_{1}^{-1}P_{2}\left(I_{2}-{\bar{Z}}_{4}^{-1}\right)\right]y\\ {\hat{\bar{x}}}^{-}={\bar{O}}^{-1}\left[\begin{array}{c} {\hat{\bar{x}}}^{{\left(1\right)}^{-}}\\ y \end{array}\right] \end{array}\right.$$

where $\ell^{+},\ell^{-}$ is the intermediate variable, ${\hat{\bar{x}}}_{1}^{{\left(1\right)}^{+}}$ ${\hat{\bar{x}}}_{1}^{{\left(1\right)}^{-}}$ are the upper and lower bounds of the observed values of ${\bar{x}}_{1}^{\left(1\right)}$, and the expression of the new adaptive approaching law $f\left({\bar{x}}^{\left(1\right)}-{\hat{\bar{x}}}_{1}^{{\left(1\right)}^{+}}\right)$ and $f\left({\bar{x}}^{\left(1\right)}-{\hat{\bar{x}}}_{1}^{{\left(1\right)}^{-}}\right)$ of the upper bound observer is

(19) $$\begin{array}{c} f\left({\bar{x}}^{\left(1\right)}-{\hat{\bar{x}}}_{1}^{{\left(1\right)}^{+}}\right)=\frac{k\tanh\left(\beta \left|{\bar{x}}^{\left(1\right)}-{\hat{\bar{x}}}_{1}^{{\left(1\right)}^{+}}\right|\right)}{1-\lambda +\lambda e^{-\alpha \left|{\bar{x}}^{\left(1\right)}-{\hat{\bar{x}}}_{1}^{{\left(1\right)}^{+}}\right|}}\\ f\left({\bar{x}}^{\left(1\right)}-{\hat{\bar{x}}}_{1}^{{\left(1\right)}^{-}}\right)=\frac{k\tanh\left(\beta \left|{\bar{x}}^{\left(1\right)}-{\hat{\bar{x}}}_{1}^{{\left(1\right)}^{-}}\right|\right)}{1-\lambda +\lambda e^{-\alpha \left|{\bar{x}}^{\left(1\right)}-{\hat{\bar{x}}}_{1}^{{\left(1\right)}^{-}}\right|}} \end{array}$$

where *k*, λ, α, and β are all normal numbers, and the constant λ satisfies 0<λ<1. Similarly, the new adaptive approach law of the lower bound observer is similar.

**Remark 4.**  *(1) According to Equation (19), when the system state is far from the sliding surface (i.e., when s is large), $\lambda e^{-\alpha \left|{\bar{x}}^{\left(1\right)}-{\hat{\bar{x}}}_{1}^{{\left(1\right)}^{+}}\right|}$ approaches 0, causing $1-\lambda +\lambda e^{-\alpha \left|{\bar{x}}^{\left(1\right)}-{\hat{\bar{x}}}_{1}^{{\left(1\right)}^{+}}\right|}$ to approach 1−λ. Due to 0<λ<1, $\frac{k}{1-\lambda }\tanh\left(\beta \left|{\bar{x}}^{\left(1\right)}-{\hat{\bar{x}}}_{1}^{{\left(1\right)}^{+}}\right|\right)$ is greater than the traditional sliding mode gain k, indicating that the system state converges quickly when it is far from the sliding surface. (2) When the system state approaches the sliding mode surface, i.e., when s is small, $\lambda e^{-\alpha \left|{\bar{x}}^{\left(1\right)}-{\hat{\bar{x}}}_{1}^{{\left(1\right)}^{+}}\right|}$ approaches 1, causing $1-\lambda +\lambda e^{-\alpha \left|{\bar{x}}^{\left(1\right)}-{\hat{\bar{x}}}_{1}^{{\left(1\right)}^{+}}\right|}$ to approach 1. At this point, $k\tanh\left(\beta \left|{\bar{x}}^{\left(1\right)}-{\hat{\bar{x}}}_{1}^{{\left(1\right)}^{+}}\right|\right)$ is less than k, indicating that chattering can be significantly suppressed as the system state approaches the sliding mode surface.*

According to Equations (17) and (18) of the reduced order interval sliding mode observer, the estimated value ${\hat{\bar{x}}}^{\left(1\right)}$ of the state variable ${\bar{x}}^{\left(1\right)}$ is written as

(20) $${\hat{\bar{x}}}^{\left(1\right)}=\eta {\hat{\bar{x}}}^{{\left(1\right)}^{-}}+\left(1-\eta \right){\hat{\bar{x}}}_{1}^{{\left(1\right)}^{+}}$$

where the weight factor $\eta =\frac{{\bar{x}}^{\left(1\right)}-{\hat{\bar{x}}}^{{\left(1\right)}^{+}}}{{\hat{\bar{x}}}^{{\left(1\right)}^{-}}-{\hat{\bar{x}}}^{{\left(1\right)}^{+}}}$ satisfies ${\hat{\bar{x}}}^{{\left(1\right)}^{-}}<{\hat{\bar{x}}}^{\left(1\right)}<{\hat{\bar{x}}}^{{\left(1\right)}^{+}}$ at any time.

Based on this foundation, we define the constant $c_{0}$ as follows:

(21) $$c_{0}=\frac{\vartheta -\vartheta^{+}}{\vartheta^{-}-\vartheta^{+}}$$

The linear combination ϖ of $\ell^{+}$ and $\ell^{-}$ is defined as

(22) $$\varpi =c_{0}{\hat{\bar{x}}}^{{\left(1\right)}^{-}}+\left(1-c_{0}\right){\hat{\bar{x}}}^{{\left(1\right)}^{+}}$$

The expression for $\dot{\varpi }$ can be obtained as follows:

(23) $$\begin{array}{c} \dot{\varpi }=\left[{\bar{A}}_{1}^{\left(2\right)}+P_{1}^{-1}P_{2}A_{3}^{\left(2\right)}\right]\varpi +\left[{\bar{A}}_{2}^{\left(2\right)}+P_{1}^{-1}P_{2}A_{4}^{\left(2\right)}\right]y+2\left[{\bar{Z}}_{2}^{-1}-P_{1}^{-1}P_{2}\left(I_{2}-{\bar{Z}}_{4}^{-1}\right)\right]\dot{y}\\ +\vartheta \hbar \left[{\bar{B}}_{1}^{\left(2\right)}+P_{1}^{-1}P_{2}{\bar{B}}_{2}^{\left(2\right)}\right]\varphi +c_{0}\left[{\bar{D}}_{1}^{\left(2\right)}+P_{1}^{-1}P_{2}{\bar{D}}_{2}^{\left(2\right)}\right]d^{-}+\left(1-c_{0}\right)\left[{\bar{D}}_{1}^{\left(2\right)}+P_{1}^{-1}P_{2}{\bar{D}}_{2}^{\left(2\right)}\right]d^{+}\\ +\left[{\bar{G}}_{1}^{\left(2\right)}+P_{1}^{-1}P_{2}{\bar{G}}_{2}^{\left(2\right)}\right]\gamma +K_{1}\left({\hat{\bar{x}}}^{\left(1\right)}-\varpi \right)+\left(1-c_{0}\right)k_{1}f\left({\bar{x}}^{\left(1\right)}-{\hat{\bar{x}}}^{{\left(1\right)}^{+}}\right)\mathrm{sign}\left({\bar{x}}^{\left(1\right)}-{\hat{\bar{x}}}^{{\left(1\right)}^{+}}\right)\\ +c_{0}k_{1}f\left({\bar{x}}^{\left(1\right)}-{\hat{\bar{x}}}^{{\left(1\right)}^{-}}\right)\mathrm{sign}\left({\bar{x}}^{\left(1\right)}-{\hat{\bar{x}}}^{{\left(1\right)}^{-}}\right) \end{array}$$

On this basis, define the error $e_{\varpi }={\bar{x}}^{\left(1\right)}-\varpi$:

(24) $$\begin{array}{c} {\dot{e}}_{\varpi }=\left[{\bar{A}}_{1}^{\left(2\right)}+P_{1}^{-1}P_{2}{\bar{A}}_{3}^{\left(2\right)}-K_{1}\right]e_{\varpi }-\left[{\bar{Z}}_{2}^{-1}-P_{1}^{-1}P_{2}\left(I_{2}-{\bar{Z}}_{4}^{-1}\right)\right]\dot{y}+{\bar{F}}_{1}^{\left(3\right)}f\\ +\left[{\bar{D}}_{1}^{\left(2\right)}+P_{1}^{-1}P_{2}{\bar{D}}_{2}^{\left(2\right)}\right]\left(d-c_{0}d^{-}-\left(1-c_{0}\right)d^{+}\right)\\ -k_{1}\left(c_{0}f\left({\bar{x}}^{\left(1\right)}-{\hat{\bar{x}}}^{{\left(1\right)}^{-}}\right)+\left(1-c_{0}\right)f\left({\bar{x}}^{\left(1\right)}-{\hat{\bar{x}}}^{{\left(1\right)}^{+}}\right)\right)\\ \cdot \left(c_{0}\mathrm{sign}\left({\bar{x}}^{\left(1\right)}-{\hat{\bar{x}}}^{{\left(1\right)}^{-}}\right)+\left(1-c_{0}\right)\mathrm{sign}\left({\bar{x}}^{\left(1\right)}-{\hat{\bar{x}}}^{{\left(1\right)}^{+}}\right)\right) \end{array}$$

To demonstrate the stability of dynamic error systems, we present the following theorem:

**Theorem 2.**  *In the case where sensor failure is not present, there exists a symmetric positive definite matrix P and Q that fulfills the following equation:*

(25) $$\begin{array}{c} {\left[{\bar{A}}_{1}^{\left(2\right)}+P_{1}^{-1}P_{2}{\bar{A}}_{3}^{\left(2\right)}-K_{1}\right]}^{T}P\\ +P\left[{\bar{A}}_{1}^{\left(2\right)}+P_{1}^{-1}P_{2}{\bar{A}}_{3}^{\left(2\right)}-K_{1}\right]=-Q \end{array}$$

*So, the error dynamic systems (23) and (24) are asymptotically stable.*

**Proof.**  Choose the following Lyapunov equation:

(26) $$V=e_{\varpi }^{T}Pe_{\varpi }$$

From Equation (26), it can be concluded that:

(27) $$\begin{array}{c} \dot{V}={\dot{e}}_{\varpi }^{T}Pe_{\varpi }+e_{\varpi }^{T}P{\dot{e}}_{\varpi }\\ =e_{\varpi }^{T}\left({\left[{\bar{A}}_{1}^{\left(2\right)}+P_{1}^{-1}P_{2}{\bar{A}}_{3}^{\left(2\right)}-K_{1}\right]}^{T}P\left.+P\left[{\bar{A}}_{1}^{\left(2\right)}+P_{1}^{-1}P_{2}{\bar{A}}_{3}^{\left(2\right)}-K_{1}\right]\right)e_{\varpi }\right.\\ -2e_{\varpi }^{T}P\left(-\left[{\bar{Z}}_{2}^{-1}-P_{1}^{-1}P_{2}\left(I_{2}-{\bar{Z}}_{4}^{-1}\right)\right]\dot{y}\right)\\ +2e_{\varpi }^{T}P\left(\left[{\bar{D}}_{1}^{\left(2\right)}+P_{1}^{-1}P_{2}{\bar{D}}_{2}^{\left(2\right)}\right]\left(d-c_{0}d^{-}-\left(1-c_{0}\right)d^{+}\right)-2e_{\varpi }^{T}P\left({\bar{F}}_{1}^{\left(3\right)}f\right)\right.\\ -2e_{\varpi }^{T}P\left(k_{1}\left(c_{0}f\left({\bar{x}}^{\left(1\right)}-{\hat{\bar{x}}}^{{\left(1\right)}^{-}}\right)+\left(1-c_{0}\right)f\left({\bar{x}}^{\left(1\right)}-{\hat{\bar{x}}}^{{\left(1\right)}^{+}}\right)\right)\right.\\ \cdot \left.\left(c_{0}sign\left({\bar{x}}^{\left(1\right)}-{\hat{\bar{x}}}^{{\left(1\right)}^{-}}\right)+\left(1-c_{0}\right)sign\left({\bar{x}}^{\left(1\right)}-{\hat{\bar{x}}}^{{\left(1\right)}^{+}}\right)\right)\right)\\ =-e_{z_{1}\varpi_{1}}^{T}Qe_{z_{1}\varpi_{1}}+2e_{\varpi }^{T}P\left(\left(-\left[{\bar{Z}}_{2}^{-1}-P_{1}^{-1}P_{2}\left(I_{2}-{\bar{Z}}_{4}^{-1}\right)\right]\dot{y}\right)\right.\\ +\left[{\bar{D}}_{1}^{\left(2\right)}+P_{1}^{-1}P_{2}{\bar{D}}_{2}^{\left(2\right)}\right]\left(d-c_{0}d^{-}-\left(1-c_{0}\right)d^{+}\right)-\left({\bar{F}}_{1}^{\left(3\right)}f\right)\\ -k_{1}\left(c_{0}f\left({\bar{x}}^{\left(1\right)}-{\hat{\bar{x}}}^{{\left(1\right)}^{-}}\right)+\left(1-c_{0}\right)f\left({\bar{x}}^{\left(1\right)}-{\hat{\bar{x}}}^{{\left(1\right)}^{+}}\right)\right)\\ \left.\cdot \left(c_{0}sign\left({\bar{x}}^{\left(1\right)}-{\hat{\bar{x}}}^{{\left(1\right)}^{-}}\right)+\left(1-c_{0}\right)sign\left({\bar{x}}^{\left(1\right)}-{\hat{\bar{x}}}^{{\left(1\right)}^{+}}\right)\right)\right) \end{array}$$

By using the Rayleigh inequality, we can obtain:

(28) $$0<\gamma_{\min}\left(Q\right){∥e_{\varpi }∥}^{2}\leq e_{\varpi }^{T}Qe_{\varpi }\leq \gamma_{\max}\left(Q\right){∥e_{\varpi }∥}^{2}$$

The minimum eigenvalue, denoted as $\gamma_{\min}\left(Q\right)$, refers to the smallest eigenvalue of a positive definite matrix *Q*. Conversely, the maximum eigenvalue, represented by $\gamma_{\min}\left(Q\right)$, indicates the largest eigenvalue of the same matrix. Additionally, the sign function, denoted as sign(), is a monotonically increasing function with a value range of $\left[-1,1\right]$. This property of the sign function ensures that as its argument increases, the output will not decrease, thereby preserving the order of the inputs within the specified range.

(29) $$\mathrm{sign}\left(e_{\varpi }\right)\leq c_{0}\mathrm{sign}\left({\bar{x}}^{\left(1\right)}-{\hat{\bar{x}}}^{{\left(1\right)}^{-}}\right)+\left(1-c_{0}\right)\mathrm{sign}\left({\bar{x}}^{\left(1\right)}-{\hat{\bar{x}}}^{{\left(1\right)}^{+}}\right)$$

Substituting Equations (28) and (29) into Equation (27) yields:

(30) $$\begin{array}{c} \dot{V}<-\gamma_{\min}\left(Q\right){∥e_{\varpi }∥}^{2}+2e_{\varpi }^{T}P\left(\left({\bar{D}}_{1}^{\left(2\right)}+P_{1}^{-1}P_{2}{\bar{D}}_{2}^{\left(2\right)}\right)\left(d-c_{0}d^{-}-\left(1-c_{0}\right)d^{+}\right)\right)\\ -2e_{\varpi }^{T}Pk_{1}f\left({\bar{x}}^{\left(1\right)}-\varpi \right)sign\left(e_{\varpi }\right)\\ <-\gamma_{\min}\left(Q\right){∥e_{\varpi }∥}^{2}+2\gamma_{\max}\left(P\right)∥e_{\varpi }∥∥\left({\bar{D}}_{1}^{\left(2\right)}+P_{1}^{-1}P_{2}{\bar{D}}_{2}^{\left(2\right)}\right)\left(d-c_{0}d^{-}-\left(1-c_{0}\right)d^{+}\right)∥\\ -2\gamma_{\max}\left(P\right)∥e_{\varpi }∥k_{1}f\left({\bar{x}}^{\left(1\right)}-\varpi \right) \end{array}$$

Using the properties of norms, write the above equation in the following form:

(31) $$\dot{V}<-\gamma_{\min}\left(Q\right){∥e_{\varpi }∥}^{2}+2\gamma_{\max}\left(P\right)∥e_{\varpi }∥\left(∥{\bar{D}}_{1}^{\left(2\right)}+P_{1}^{-1}P_{2}{\bar{D}}_{2}^{\left(2\right)}∥\left(d^{-}+3d^{+}\right)-k_{1}\cdot f\left({\bar{x}}^{\left(1\right)}-\varpi_{1}\right)\right)$$

To ensure $\dot{V}<0$, there must be:

(32) $$k_{1}\cdot f\left({\bar{x}}^{\left(1\right)}-\varpi_{1}\right)>∥{\bar{D}}_{1}^{\left(2\right)}+P_{1}^{-1}P_{2}{\bar{D}}_{2}^{\left(2\right)}∥d^{-}+3∥{\bar{D}}_{1}^{\left(2\right)}+P_{1}^{-1}P_{2}{\bar{D}}_{2}^{\left(2\right)}∥d^{+}$$

Under normal conditions, in order for the error between the reduced order interval sliding mode observer and the actual rectifier system to converge to 0 in finite time, $\dot{V}V<0$ is still required

(33) $$\dot{V}<-k_{1}∥e_{\varpi }∥$$

From Equation (33), we can obtain $\sqrt{V}=∥e_{\varpi }∥$. Substituting it into the above equation, we can obtain:

(34) $$\dot{V}<-k_{1}\sqrt{V}$$

Building on the preceding analysis, it can be concluded that $\dot{V}<0$ implies the error between the observed values from the normal state descending order interval sliding mode observer and the actual system will converge to zero within a finite time. This outcome suggests that the system exhibits asymptotic stability. □

**Remark 5.**  *The design of the reduced-order sliding mode observer offers significant flexibility, as it imposes no restrictions on the types or occurrence times of current sensor faults. Furthermore, the proposed observer demonstrates the capability to estimate any type of current sensor fault at any given moment, ensuring robust fault detection and diagnosis in varying conditions.*

### 3.3. Fault Detect

The state estimation for the LLC resonant converter system can be derived using the reduced-order interval sliding mode observer. The specific algorithm for this estimation is presented as follows:

(35) $$\begin{array}{c} x^{+}=\left[\begin{array}{cc} O_{1}^{-1} & O_{2}^{-1} \end{array}\right]\left[\begin{array}{c} {\hat{\bar{x}}}_{1}^{{\left(1\right)}^{+}}\\ y \end{array}\right],x^{-}=\left[\begin{array}{cc} O_{1}^{-1} & O_{2}^{-1} \end{array}\right]\left[\begin{array}{c} {\hat{\bar{x}}}_{1}^{{\left(1\right)}^{-}}\\ y \end{array}\right]\\ {℘}^{+}=\left[\begin{array}{cc} O_{3}^{-1} & O_{4}^{-1} \end{array}\right]\left[\begin{array}{c} {\hat{\bar{x}}}_{1}^{{\left(1\right)}^{+}}\\ y \end{array}\right],{℘}^{-}=\left[\begin{array}{cc} O_{3}^{-1} & O_{4}^{-1} \end{array}\right]\left[\begin{array}{c} {\hat{\bar{x}}}_{1}^{{\left(1\right)}^{-}}\\ y \end{array}\right] \end{array}$$

Fault detection variables have been created based on the upper and lower limits derived from the reduced-order sliding mode observer. Additionally, adaptive diagnostic thresholds have been established to support the fault detection process.

(36) $$\kappa =\frac{2i\left(t\right)-i^{+}\left(t\right)-i^{-}\left(t\right)}{2}$$

Based on the fault analysis presented above, if a malfunction occurs in either the power switch or the current sensor, the actual output current $i_{o}\left(t\right)$ of the LLC resonant converter will become distorted. This distortion causes the fault detection variable κ to deviate from zero.

Leveraging the interval characteristics of the reduced-order sliding mode observer, adaptive thresholds for fault detection variables have been developed. Specifically, upper adaptive threshold th1 and lower adaptive threshold th2 have been established.

(37) $$\left\{\begin{array}{c} th_{\kappa }^{+}=i^{+}\left(t\right)-i\left(t\right)\\ th_{\kappa }^{-}=i^{-}\left(t\right)-i\left(t\right) \end{array}\right.$$

From Equations (36) and (37), it can be seen that when the LLC resonant converter system does not experience a fault, κ is approximately 0, $Th_{\kappa 1}\;>0$, $Th_{\kappa 2}<0$, that is, κ is located between $Th_{\kappa 1}$ and $Th_{\kappa 2}$. However, if the LLC resonant converter system encounters an open circuit fault in the rectifier switch or a fault in the current sensor, this may lead to variations in the fault detection variable κ as well as the adaptive thresholds $Th_{\kappa 1}$ and $Th_{\kappa 2}$. When κ is no longer between the adaptive thresholds $Th_{\kappa 1}$ and $Th_{\kappa 2}$, a fault in the LLC resonant converter system can be detected. Simultaneously, the fault detection variable κ and the adaptive thresholds $Th_{\kappa 1}$ and $Th_{\kappa 2}$ adjust according to the state of the LLC resonant converter. This adaptive behavior helps prevent false alarms triggered by normal operating conditions, such as fluctuations in load.

### 3.4. Fault Identification

To effectively identify open circuit faults in inverter switch tubes and faults in current sensors, it is essential to differentiate between two distinct fault types. Consequently, utilizing the estimated upper and lower bounds of the current sensor fault derived from the formula, the estimated value of the current sensor fault can be defined as:

(38) $$\hat{℘}\left(t\right)=\alpha {℘}^{+}\left(t\right)+\left(1-\alpha \right){℘}^{-}\left(t\right)$$

Among them, appropriate constants between $\alpha \in \left(0,1\right)$. Sensor fault identification is achieved by analyzing Equation (38), which indicates that when a current sensor fails, the observed value satisfies $\hat{℘}\left(t\right)\neq 0$. In contrast, for an open-circuit fault in the inverter switching device, the condition is represented by $\hat{℘}\left(t\right)=0$. Thus, the estimated value of the sensor fault can serve as a criterion to differentiate between open-circuit faults in the inverter switching device and faults in the current sensor. Additionally, to enhance the accuracy and robustness of the fault identification process, when $\hat{℘}\left(t\right)\in \left[-\zeta ,\zeta \right]$ is satisfied, $\hat{℘}\left(t\right)=0$, where ζ is set as 5% of the current amplitude before the fault. Based on the above analysis, the identification of open circuit faults in rectifier switch tubes and current sensor faults is as follows:

(39) $$\Delta_{x}=\left\{\begin{array}{c} \mathrm{OC},\hat{℘}\in \left[-\zeta ,\zeta \right]\\ \mathrm{CS},\hat{℘}\notin \left[-\zeta ,\zeta \right] \end{array}\right.$$

When $\Delta_{x}=OC$ indicates an open circuit fault in the rectifier switch tube, $\Delta_{x}=CS$ indicates a current sensor fault.

### 3.5. Fault Power Switch Tube Positioning

On the basis of detecting open circuit faults in LLC resonant converters, this section analyzes the distortion characteristics of the output current waveform and designs two fault localization variables based on current residuals by using the residual between the estimated current value of the observer under fault conditions and the normal output current value to achieve open circuit fault localization in LLC resonant converters.

The impact of open-circuit faults in the topology of an LLC resonant converter is significant. When an open-circuit fault occurs in either the bridge arm phase *a* or the bridge arm phase *b*, the actual output current waveform will attenuate, resulting in a positive residual between the estimated current and the actual current. Conversely, if an open-circuit fault occurs in the bridge arm phase *a* or phase *b*, comparing the actual output current waveform with the estimated current provided by the observer will yield a negative residual.

Building on the previous analysis, this paper employs the distinct current distortion patterns associated with open circuit faults in switching tubes, as well as in the upper and lower bridge arm switching tubes, to establish an initial positioning metric derived from current residuals. The formulation is presented as follows:

(40) $$\vartheta =\mathrm{sgn}{\left(\hat{i}\left(t\right)-i\left(t\right)\right)}_{avg}$$

From the examination of faults in power switch tubes, the correlation between the switching function and the switch state during the open circuit fault of phase *a* switch tube $S_{a1}$ is illustrated in Table 1:

Logical operations can be performed on Table 1 to obtain the *a* phase switching function in the open-circuit fault state of $S_{k1}$:

(41) $$\varepsilon_{ab}^{k1*}=\bar{\delta }\left(\mu_{k1}{\bar{\mu }}_{k2}+{\bar{\mu }}_{k1}{\bar{\mu }}_{k2}\right)$$

Similarly, the *a* phase switching function under the $S_{k2}$ open-circuit fault state can be derived:

(42) $$\varepsilon_{ab}^{k2*}=\delta \left(\mu_{k1}{\bar{\mu }}_{k2}+{\bar{\mu }}_{k1}{\bar{\mu }}_{k2}\right)+\bar{\delta }$$

According to the established state space model, the output current depends on the switching signal $\varepsilon_{ab}^{*}$. If the a-phase switching function $\varepsilon_{ab}$ in the normal state is replaced with the switching function $\varepsilon_{ab}^{*}$ of the a-phase in the switching fault state, the LLC resonant converter state space model in the fault state can be obtained:

(43) $$\left\{\begin{array}{c} {\dot{x}}^{*}=Ax^{*}+\hbar^{*}B^{*}\varphi^{*}+G\gamma^{*}\\ y=Cx^{*} \end{array}\right.$$

where $x^{*}=\left[\begin{array}{c} i_{o}^{*}\\ u_{C_{0}}^{*} \end{array}\right]$, $\varphi^{*}=\left[\begin{array}{c} V_{in}^{*}\\ {\frac{di_{r}}{dt}}^{*}\\ u_{r}^{*} \end{array}\right]$, $\gamma^{*}=\left[\begin{array}{c} \left|i_{p}^{*}\right|\\ V_{o}^{*} \end{array}\right]$, $\hbar^{*}=\frac{1}{n\mathrm{sgn}\left(i_{p}^{*}\right)}$, $B^{*}=\left[\begin{array}{ccc} \frac{\varepsilon_{ab}^{*}}{L_{o}} & -\frac{L_{r}}{L_{o}} & -\frac{1}{L_{o}}\\ -\frac{\varepsilon_{ab}^{*}}{C_{0}R_{L}} & \frac{L_{r}}{C_{0}R_{L}} & \frac{1}{C_{0}R_{L}} \end{array}\right]$.

By utilizing the distortion characteristics of the output current of the converter, a fault detection variable *r* based on the root mean square of the absolute value of the current is constructed, and its formula is:

(44) $$\varsigma =\frac{\log_{\tau }\left(1+{\left|i_{o}\left(t\right)\right|}_{RMS}\right)\ln\tau }{{\left|i_{o}\left(t\right)\right|}_{RMS}}$$

Among them, τ>1 introduces RMS to reduce the impact of peak current and current harmonics.

On this basis, using the estimated current of the observer and the actual output current, the adaptive threshold of the measured variable for open circuit faults is designed as follows:

(45) $$TH=\frac{\log_{\tau }\left(1+\left|{\left|{\hat{i}}_{o}\left(t\right)\right|}_{RMS}-{\left|i_{o}\left(t\right)\right|}_{RMS}\right|\right)\ln\tau }{\left|{\left|{\hat{i}}_{o}\left(t\right)\right|}_{RMS}-{\left|i_{o}\left(t\right)\right|}_{RMS}\right|}$$

To further locate the specific faulty switch tube, while ensuring that there are no false alarms in fault detection, within the fundamental wave period after completing the preliminary positioning of the switch tube, the derived switch function $\varepsilon_{ab}^{*}$ of the a-phase switch tube in the $S_{a1}$ open circuit fault state is used to replace the switch function in the normal state to design a reduced order interval sliding mode observer in the fault state. The estimated current of the observer in the $S_{a1}$ fault state is obtained, and then the fault positioning variable and positioning threshold are designed based on the estimated fault current to achieve positioning, that is

(46) $$S_{kj}=\left\{\begin{array}{c} S_{a},\varsigma <TH\\ S_{b},\varsigma >TH \end{array}\right.$$

By combining Equations (44)–(46), the fault power switch of the LLC resonant converter can be located, which can be represented by the following equation:

(47) $$\begin{array}{c} S_{aj}=\left\{\begin{array}{c} S_{a1},\vartheta =-1\&\varsigma <T_{th}\\ S_{a2},\vartheta =1\&\varsigma <T_{th} \end{array}\right.\\ S_{bj}=\left\{\begin{array}{c} S_{b1},\vartheta =1\&\varsigma >T_{th}\\ S_{b2},\vartheta =-1\&\varsigma >T_{th} \end{array}\right. \end{array}$$

In the formula, $S_{aj},S_{bj}$ represents the open-circuit fault of the switching tubes in phase a and phase b, respectively.

## 4. Simulation Example

**Figure 7:**
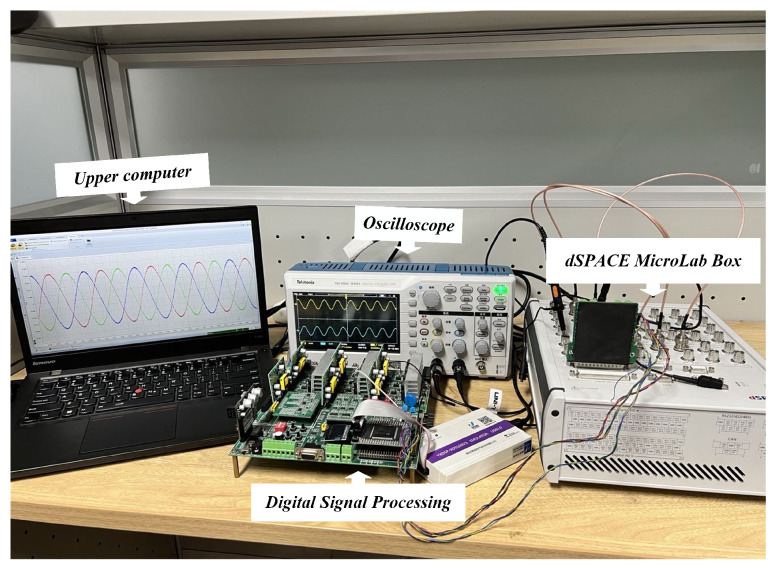
Hardware in the loop experimental device.

This study employs a hardware-in-the-loop (HIL) simulation setup to assess the effectiveness and robustness of the fault diagnosis method. As shown in Figure 7, The simulation equipment includes a dSPACE simulator, the device model is: MicroLabBox 1202/1302, Produced in Germany, a host PC, a Digital Signal Processor (DSP) model TMS320F28335, and an oscilloscope. Key parameters for the DC charging pile module are detailed in Table 2 below.

### 4.1. Minor Fault Diagnosis of Current Sensor

This section presents open-circuit (OC) faults and current sensor (CS) faults in LLC resonant converter systems to illustrate the efficacy of the proposed fault diagnosis approach.

**Figure 8:**
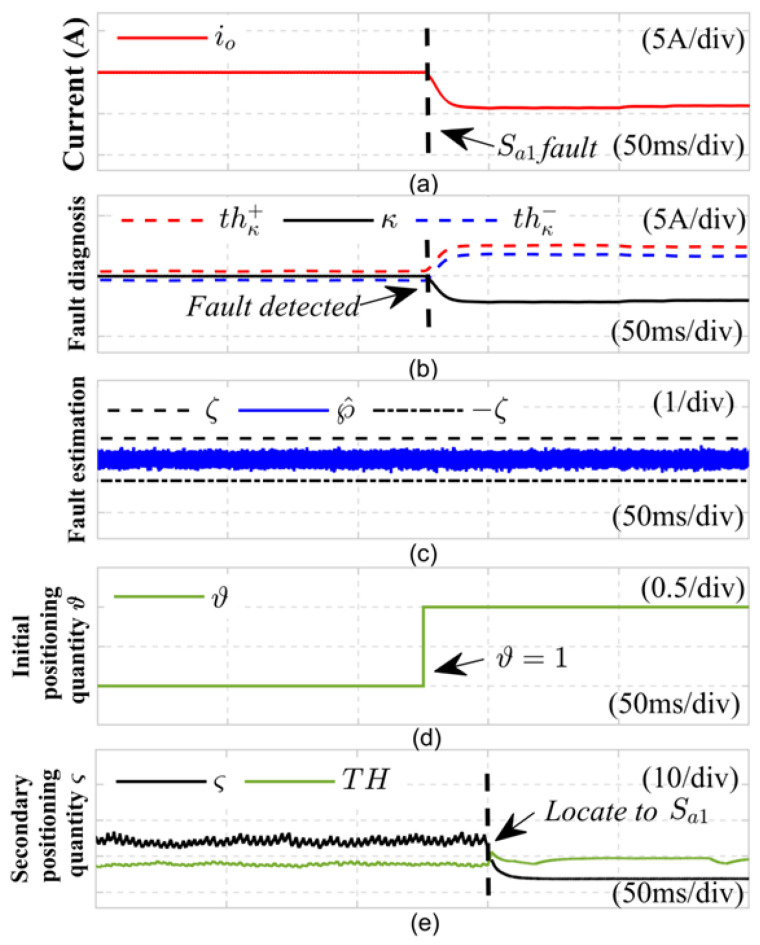
Diagnosis results of open circuit fault of power switch tube $S_{a2}$.

**Figure 9:**
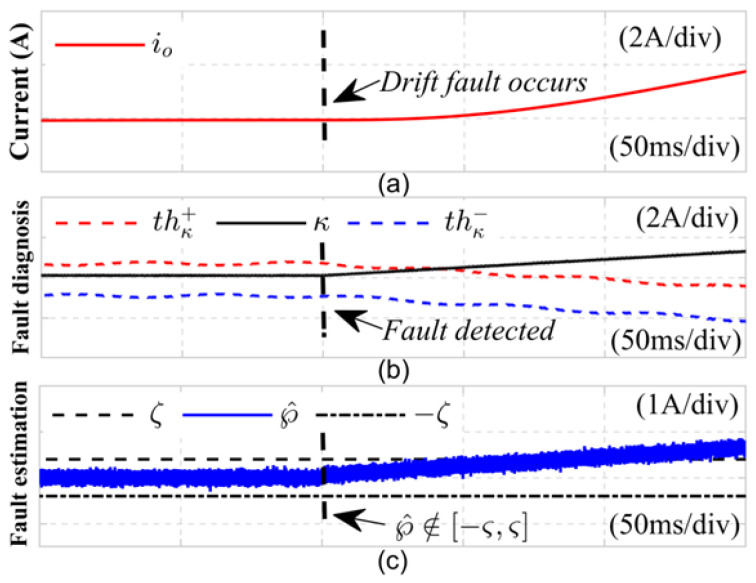
Drift fault diagnosis results of DC side current sensor.

(1) Open Circuit Fault in the Switching Tube: The diagnostic outcomes for the $S_{a2}$ switching tube in the *a* phase of the LLC resonant converter are depicted in Figure 8 during an open circuit fault. In this experiment, at *t* = 0.15 s, the pulse control signal from the DSP to the *a* phase bridge arm switching tube was interrupted, simulating an open circuit fault in $S_{a2}$. As illustrated in Figure 8a, prior to the fault, the actual DC side current remained steady at 22A. As illustrated in Figure 8b, the upper and lower limits of the reduced-order sliding mode observer were utilized to establish a fault detection variable that stayed stable within the detection thresholds $th_{\kappa }^{+}$ and $th_{\kappa }^{-}$. However, when *t* = 0.15 s, the power switch tube open circuit fault occurred, and the actual DC side current waveform was severely distorted. The fault detection quantity κ was no longer within the adaptive threshold $th_{\kappa }^{+},th_{\kappa }^{-}$ interval after the fault occurred, and the detection time was 1 ms. The estimated fault value of the current sensor satisfies $\hat{℘}\left(t\right)\in \left[-\zeta ,\zeta \right]$, indicating that the system has experienced an OC fault. At the same time, from Figure 8c, it can be seen that the initial positioning quantity ϑ switches to 1, indicating that $S_{a2}$ or $S_{b1}$ has an open-circuit fault. Subsequently, at 1/2 of the fundamental cycle time after completing the initial positioning of the switch tube, the switch function $\delta_{ab}$ in the normal state is replaced with the switch function $\varepsilon_{ab}^{k2*}$ in the $S_{a2}$ fault state, and an adaptive sliding mode observer was established for the fault state of $S_{a2}$. As illustrated in Figure 8d, the fault positioning variable ς falls below the positioning threshold TH, confirming that the *a* phase switch tube has experienced an open circuit fault. Therefore, as shown in Figure 8e, it can be concluded that Equation (47) effectively diagnoses the open circuit fault in switch tube $S_{a2}$.

(2) To evaluate the effectiveness of the proposed joint fault diagnosis method for common current sensor (CS) faults, two classic types of CS faults were examined: drift faults and bias faults. Initially, Figure 9 presents the diagnostic results related to the drift fault on the DC side current sensor, where the fault current for the drift condition is expressed as follows: $i_{f}\left(t\right)=\left\{\begin{array}{c} i\left(t\right),0\leq t\leq t_{f}\\ i\left(t\right)+K_{2}\left(t-t_{f}\right),t\geq t_{f} \end{array}\right.$. In the experiment, as shown in subgraph (a), the DC side current sensor experienced a drift fault within 0.24 s, with a drift coefficient of $K_{2}=25$, resulting in severe distortion of the DC side load current. At this point, the fault detection variable kappa in subgraph (b) rapidly increases, surpassing the detection thresholds $th_{\kappa }^{+}$ and $th_{\kappa }^{-}$ at 0.241 s. At the same time, in subgraph (c), the disturbance sensor fault estimation value $\hat{℘}\left(t\right)$ exceeds the empirical threshold range $\left[-\zeta ,\zeta \right]$, indicating that the current sensor has malfunctioned; this signifies the identification of a DC side current sensor fault within the LLC resonant converter system, with the fault being detected in just 1 ms.

**Figure 10:**
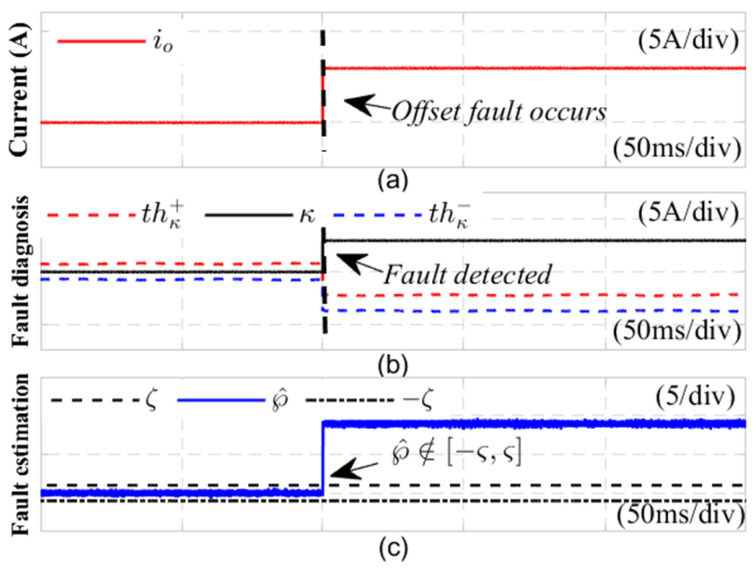
Diagnosis results of DC side current sensor offset fault.

Figure 10 shows the diagnostic results of the DC side CS bias fault, and the fault current expression with bias fault is represented as $i_{f}\left(t\right)=\left\{\begin{array}{c} i\left(t\right),0\leq t\leq t_{f}\\ i\left(t\right)+K_{1},t\geq t_{f} \end{array}\right.$. In the experiment, as shown in subgraph (a), a gain fault was detected at 0.232 s, with a gain coefficient $K_{1}$ of 6. It is clear that, in subgraph (b), upon the occurrence of this gain fault, the fault detection variable κ exceeds the adaptive thresholds $th_{\kappa }^{+}$ and $th_{\kappa }^{-}$ at 0.233 s. Concurrently, in subgraph (c), the estimated disturbance sensor fault value, $\hat{℘}\left(t\right)$, surpasses the empirical threshold range $\left[-\zeta ,\zeta \right]$, indicating a malfunction of the current sensor at *t* = 0.232 s. This result confirms the identification of the DC side current sensor fault, achieved with a rapid detection time of just 1 ms. Together, these results illustrate that the proposed method is effective in diagnosing current sensor faults and demonstrates a high diagnostic speed.

### 4.2. Robustness Verification

(1) DC side voltage fluctuation

**Figure 11:**
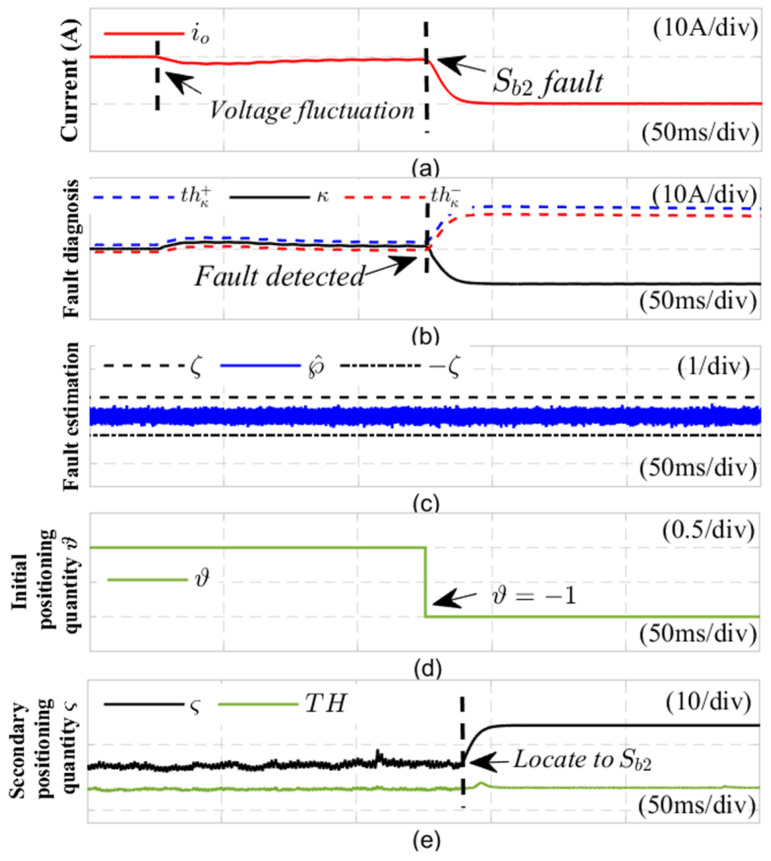
Robustness verification results of $S_{b2}$ open circuit fault under DC voltage fluctuation fluctuations.

The evaluation of the fault detection method is illustrated in Figure 11, which presents the diagnostic results for an open-circuit fault in the power switch $S_{b2}$ under conditions of DC-side voltage fluctuations. As shown in subgraph (a), a voltage fluctuation is introduced at 0.16 s, leading to an open-circuit fault in the power switch $S_{b2}$ at 0.20 s. During the voltage fluctuation at 0.16 s, the output current experiences slight disturbances; however, in subgraph (b), the fault detection variable κ remains within the adaptive threshold range, preventing any false alarms. When the current sensor fault occurs at 0.20 s, the fault detection variable κ exceeds the detection threshold $th_{\kappa }^{-}$, with a detection time of 1 ms. In subgraph (c), the estimated current sensor fault value satisfies $\hat{℘}\left(t\right)\in \left[-\zeta ,\zeta \right]$, indicating that the system has experienced an open-circuit fault (OC). At the same time, the initial positioning variable ϑ switches to −1, indicating that $S_{a1}$ or $S_{b2}$ has an open circuit fault. Subsequently, at 1/2 of the fundamental cycle time after completing the initial positioning of the switch tube, the switch function $\delta_{ab}$ in the normal state is replaced with the switch function $\varepsilon_{ab}^{k1*}$ in the fault state $S_{a1}$, and an adaptive sliding mode observer is implemented in the fault state $S_{a1}$. As shown in Figure 11d, the value of the initial positioning metric ϑ becomes −1, and at the same time, subfigure (e) shows that the second positioning variable ς did not exceed the corresponding threshold TH at the time of the fault occurrence, confirming that the phase *b* switch tube has an open circuit fault. This indicates that the fault detection method introduced in this study is not affected by DC side voltage fluctuations and successfully identifies open circuit faults in the switching tube.

(2) Sudden change in load parameters

**Figure 12:**
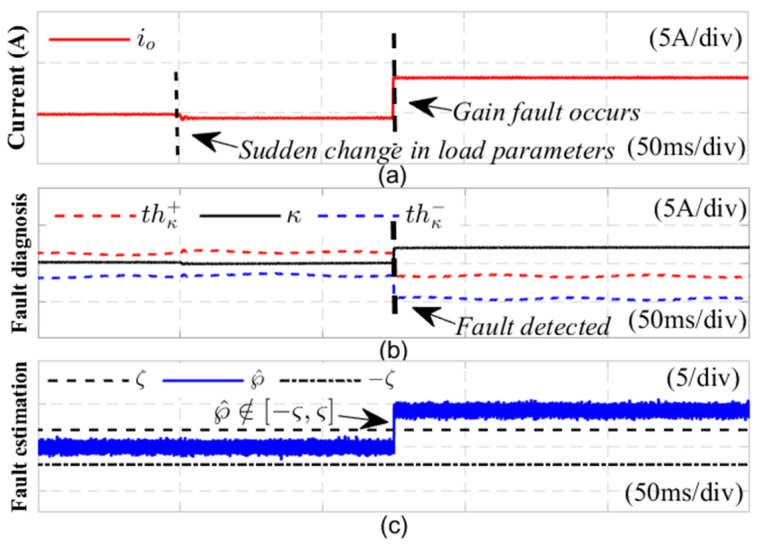
Verification results of gain fault robustness under sudden changes in DC side load parameters.

In order to evaluate the robustness of the fault detection method introduced in this study to load fluctuations, Figure 12 shows the diagnostic results of the current sensor gain fault when the load resistance suddenly changes. At 0.21 s, the load increased by 1.2 times, and a current sensor gain fault occurred at 0.28 s. In subgraph (a), after the load fluctuates, the output current fluctuates slightly, resulting in a corresponding change in the fault detection variable κ in subgraph (b). Due to the fact that κ is constructed based on the upper and lower bounds of the reduced order interval sliding mode observer, when the load fluctuates significantly, the output voltage of the system will change accordingly, and the upper and lower bounds of the observer will also fluctuate accordingly. This causes the fault detection variable κ to exhibit slight fluctuations during the load change process. However, the fault detection variable κ remained between the fault detection thresholds $th_{\kappa }^{+}$ and $th_{\kappa }^{-}$, without exceeding the upper and lower bounds, thus no fault false alarms occurred. This indicates that although the current and voltage of the system have changed, load fluctuations will not affect the accuracy of fault diagnosis.

When the current sensor malfunctions at 0.28 s, Figure 12b shows that the fault detection variable κ exceeds the upper limit th+k of the adaptive threshold range, Meanwhile, Figure 12c shows that the fault distinguishing variable $\hat{℘}$ has undergone a sudden change and is no longer within the $\left[-\zeta ,\zeta \right]$ interval, indicating that the current sensor has malfunctioned and triggering a fault alarm. This process validated the robustness of the fault diagnosis method under load fluctuations, successfully distinguishing between load changes and current sensor faults, ensuring the stable operation of the system.

### 4.3. Performance Comparison

We sincerely thank everyone for their constructive opinions and suggestions on this matter. Based on your valuable feedback, a comparison between previously reported methods and suggested methods has been added in the performance comparison section of Section 4.3 from several aspects. Meanwhile, for the sake of clarity, the details of the newly added Performance Comparison section are further introduced as follows.

To demonstrate the effectiveness of the developed fault diagnosis method, a comparison was made with previously reported methods for diagnosing faults in LLC resonant converters and similar rectifiers. Table 3 shows the comparison results. In previous methods [12,18,27], the impact of CS faults on IGBT OC fault detection methods was not considered. In addition, refs. [23,28] focused more on CS faults. In contrast, the method proposed in this paper can simultaneously diagnose IGBT OC faults and various types of CS faults.

In addition, the proposed method relies solely on three-phase current for fault diagnosis, while existing methods [27] use both bridge arm midpoint voltage and three-phase current to diagnose IGBT OC faults, requiring additional sensors to measure terminal voltage. In addition, previous methods [12,18,27] had relatively long fault detection times. In contrast, the proposed method can quickly detect faults with a detection time of about 1 ms. Meanwhile, another drawback of the previously reported method [28] is that it uses a fixed threshold for fault detection, resulting in poor robustness. In contrast, the proposed method adopts adaptive thresholding to enhance the robustness of fault detection. Overall, the proposed method can effectively diagnose IGBT OC faults and various types of CS faults, with characteristics such as short detection time, strong robustness, moderate algorithm complexity, etc., without the need for additional hardware and large amounts of data. Through verification, comparison, and analysis, it can be concluded that the developed fault diagnosis method is highly suitable for effectively detecting and diagnosing MOSFET OC and CS faults used in LLC resonant converters.

## 5. Conclusions

This paper presents an innovative fault diagnosis method that utilizes a reduced-order interval sliding mode observer (SMO) to simultaneously diagnose open-circuit (OC) faults and current sensor (CS) faults within the DC charging pile module. The underlying principle of this method involves the effective integration of state augmentation and the application of matrix transformations, resulting in a design that can accurately estimate both phase currents and current sensor faults concurrently. By decoupling the estimation results of the system, this approach enables the identification of both current sensor faults and open-switch faults, thus facilitating the localization of the faulty power switch and the estimation of current sensor issues. The design significantly enhances the accuracy and robustness of the fault diagnosis process, allowing for dynamic threshold adjustments that enable the method to adapt flexibly to varying operational conditions for fault detection. Additionally, this approach effectively reduces the likelihood of misjudgments caused by environmental changes or noise interference, showcasing a higher level of reliability and stability in practical applications. In summary, the proposed joint fault diagnosis method for OC and CS faults in the DC charging station charging module based on reduced-order interval SMO effectively diagnoses OC and CS faults, providing strong guarantees for the safe operation of DC charging stations.

Future work will expand the fault model to incorporate additional component stresses, including thermal effects and load variations, to further improve the system’s robustness and adaptability. Additionally, we aim to explore multi-fault diagnosis mechanisms that can jointly detect multiple faults, improving diagnostic accuracy by cross-validating fault characteristics and reducing the likelihood of misdiagnosis. These enhancements will contribute to a more comprehensive and reliable fault diagnosis framework, better suited for real-world applications and complex operating conditions in DC charging stations. 

## Figures and Tables

**Table 1:** The relationship between switch function and gate signal, current direction.

$\mu_{k1}$	$\mu_{k2}$	$\varepsilon \left(\delta =1\right)$	$\varepsilon \left(\delta =0\right)$
0	0	0	1
1	1	0	0
1	0	0	1

**Table 2:** Main parameters of LLC Resonant Converter.

Pre Set Indicators	Parameter
Original secondary winding turns ratio	2.5
Switching frequency of power devices	90∼180 kHz
Output power	4400 W
filter capacitor	500 µF
Output current	22A
Resonant inductor	40 µH
filter inductance	44 nH
Output voltage	200 V

**Table 3:** Performance comparison with relevant approaches.

Relevant Diagnosis Approach	Fault Types	Parameter	Detection Time for Open-Circuit Fault	Complexity of Diagnosis Approach
[12]	Open-circuit fault	Phase voltage	0.5–3 FP	Medium
[18]	Open-circuit fault	Resonant capacitor voltage	<3 FP	Medium
[27]	Open-circuit fault	Midpoint voltage of the bridge arm	≈2 FP	Medium
[28]	Current sensor fault	Phase Current	>0.5 FP	High
[23]	Current sensor & position sensor fault	Dq-axi Current	≈0.5 FP	High
This method	Open circuit fault & Current sensor fault	Phase Current	≈0.1 FP	Medium

## Data Availability

Data are contained within the article.
